# The Bristol Medical Dramatic Club

**Published:** 1937

**Authors:** 


### THE BRISTOL MEDICAL DRAMATIC CLUB.

The following table is published as an addendum to the brief history of the Bristol Medical Dramatic Club which occurred in our last issue.

Since then the Editor has received invaluable help from communications kindly sent by Col. James Swain and Mr. F. Lace, and he wishes to take this opportunity of expressing his gratitude to these past pioneers, also to those who have furnished many useful records, especially Dr. H. R. Husbands, Dr. P. Watson-Williams, Mrs. J. C. Heaven, and Dr. D. Munro Smith.

Space does not permit us to include the names of members and friends who have been so largely responsible for the Club's prosperity by their efforts as members or officers of the General and Ladies' Committees, or of those who have so generously helped in the orchestra. Many valuable records were lost in Crichton's fire in 1897, but private albums and newspaper files have supplied most of the missing details.

The numerals in the table indicate the number of sessions in which each individual has appeared in some capacity or other, and date of first association with the Club is also shown.

| | Acted. | Stage Manager or Producer. | President. | Date. |
|---|---|---|---|---|
| A | | | | |
| Avery, Miss Maud .. | .. | .. | .. | 1881 |
| Arnold, C. .. .. .. | — | 2 | — | 1882 |
| Alford, E. H. M. .. | 1 | — | — | 1884 |
| Austin, R. .. .. .. | 1 | — | — | 1890 |
| Ackland, W. R. .. .. | 1 | — | 2 | 1891 |
| Austin, Miss Norah .. | 1 | — | — | 1898 |
| Anson, L. .. .. .. | 1 | — | — | 1898 |
| Allen, Miss .. .. .. | 1 | — | — | 1901 |
| Adcock, J. P. .. .. | 1 | — | — | 1911 |
| Archbold, Mrs. .. .. | 1 | — | — | 1912 |
| Armstrong, Miss J. E. | 1 | — | — | 1927 |
| Arthur, R. .. .. .. | 1 | — | — | 1929 |
| Atchison, Miss E. .. | 2 | — | — | 1930 |
| B | | | | |
| Bruce, Gerald .. .. | 1 | — | — | 1881 |
| Bramhall, Miss .. .. | 8 | — | — | 1882 |
| Barnes, Miss Alice .. | 2 | — | — | 1882 |
| Begbie, F. W. .. .. | 2 | — | — | 1883 |
| Baskett, B. .. .. .. | 1 | — | — | 1888 |
| Bernard, C. .. .. .. | 2 | — | — | 1889 |
| Beresford, E. H. .. | 1 | — | — | 1889 |
| Bate, J. G. .. .. .. | 1 | — | — | 1890 |
| Barclay, Wm. .. .. | 1 | — | — | 1892 |
| Bridges, Victor .. .. | 1 | — | — | 1900 |
| Beloe, Miss Evelyn .. | 1 | — | — | 1900 |
| Brickdale, Fortescue .. | 1 | — | — | 1904 |
| Board, J. H... .. .. | 1 | — | — | 1905 |
| Beilby, Miss .. .. .. | 1 | — | — | 1907 |
| Buckle, Miss .. .. .. | 3 | — | — | 1910 |
| Bush, G. B. .. .. .. | 4 | — | — | 1920 |
| Buchan, H. F. .. .. | 2 | — | — | 1920 |
| Butler, Miss E. B. .. | 1 | — | — | 1923 |
| Bulleid, J. H. .. .. | 2 | — | — | 1930 |
| Brown, W. S. A. .. .. | 2 | — | — | 1935 |
| Bradbeer, Mrs. S. G. .. | 2 | — | — | 1935 |
| Buret, Miss V. .. .. | 1 | — | — | 1936 |
| Bain, Mrs. .. .. .. | 1 | — | — | 1936 |
| C | | | | |
| Collins, Miss Marie .. | 2 | — | — | 1880 |
| Cross, F. R. .. .. .. | — | — | 3 | 1888 |
| Chilton, R. H. .. .. | 2 | — | — | 1892 |
| Clarke, J. Michell .. | — | — | 2 | 1893 |
| Clarke, E. H. .. .. | 1 | — | — | 1893 |
| Cridland, A. B. .. .. | 5 | — | — | 1894 |
| Clayton, J. C. .. .. | 3 | — | — | 1896 |
| Carter, Miss Margaret | 2 | — | — | 1898 |
| Corfield, C. .. .. .. | 2 | — | — | 1901 |
| Cridland, Miss .. .. | 3 | — | — | 1903 |
| Chilton, Alan .. .. | 1 | — | — | 1903 |
| Conolly, N. .. .. .. | 1 | — | — | 1904 |
| Chitty, H. .. .. .. | 5 | — | — | 1910 |
| Charlton, Miss Maud .. | 1 | — | — | 1913 |
| Clarke, R. C. .. .. | 1 | 4 | — | 1914 |
| Critchley, M. .. .. | 3 | — | — | 1920 |
| Charles, J. R. .. .. | — | — | 1 | 1923 |
| Crellin, Miss D. .. .. | 1 | — | — | 1924 |
| Collinson, Miss E. M. D. M. | 7 | — | — | 1925 |
| Cory, R. A. S. .. .. | 4 | — | — | 1926 |
| Corfield, H. F. G. .. | 4 | — | — | 1926 |
| Coates, J. F. .. .. | 2 | — | — | 1929 |
| Coulter, J. L. S. .. | 2 | — | — | 1929 |
| Coombs, N. C. .. .. | 1 | — | — | 1929 |
| Claremont, L. E. .. | 1 | — | — | 1936 |
| Cossens, Miss .. .. | 1 | — | — | 1936 |
| D | | | | |
| Dunn, G. .. .. .. | 1 | — | — | 1881 |
| Dobbie, Miss .. .. .. | 1 | — | — | 1883 |
| Dykes, P. A. .. .. .. | 3 | — | — | 1889 |
| Davies, Mrs. .. .. .. | 1 | — | — | 1892 |
| Davies, D. S. .. .. | — | 8 | — | 1895 |
| Day, Miss .. .. .. | 1 | — | — | 1904 |
| Dacre, R. I. .. .. .. | 3 | — | — | 1909 |
| Davies, E. D. .. .. | 1 | — | — | 1910 |
| Derry, B. G. .. .. | 2 | — | — | 1911 |
| Dixon, H. M. .. .. | 2 | — | — | 1920 |
| Drew, Miss M. D... .. | 3 | — | — | 1921 |
| Davis, C. J. N. .. .. | 2 | — | — | 1928 |
| Derham, Miss R. M. S. | 2 | — | — | 1929 |
| Denning, G. M. .. .. | 2 | — | — | 1930 |
| Davies, J. L. W. .. .. | 1 | — | — | 1931 |
| Dymond, D. J. .. .. | 1 | — | — | 1936 |
| Dennis, Miss .. .. .. | 1 | — | — | 1936 |
| E | | | | |
| Edwards, G. Dundas .. | 3 | 2 | — | 1888 |
| Eyre, S. P. .. .. .. | 1 | — | — | 1906 |
| Edwards, A. C. .. .. | 1 | — | — | 1926 |
| Ernest, John .. .. | 1 | — | — | 1928 |

| | Acted. | Stage Manager or Producer. | President. | Date. |
|---|---|---|---|---|
| **F** | | | | |
| Fairchild, Miss Lucy .. | 1 | — | — | 1884 |
| Fowler, C. E. P. .. .. | 1 | — | — | 1884 |
| Fardon, J. H. .. .. | 1 | — | — | 1888 |
| Foote, Miss .. .. .. | 1 | — | — | 1894 |
| Fawcett, Mrs. E. .. .. | 2 | — | — | 1895 |
| Flemming, A. L. .. .. | 2 | — | 10 | 1904 |
| Fawcett, A. .. .. .. | 1 | — | — | 1910 |
| France-Hayhurst, Miss C. | 2 | — | — | 1921 |
| Fox, Miss C. F. .. .. | 4 | — | — | 1922 |
| Forsyth, W. .. .. .. | 1 | — | — | 1929 |
| Fosbery, F. W. A. .. | 1 | — | — | 1929 |
| Frossard, Mrs. .. .. | 1 | — | — | 1935 |
| **G** | | | | |
| Glasson, C. .. .. .. | 1 | — | 2 | 1882 |
| Gibbs, A. N. Godby .. | 2 | — | — | 1884 |
| Girdlestone, Miss Gladys | 2 | — | — | 1907 |
| Gordon, Miss M. .. .. | 2 | — | — | 1910 |
| Gaskell, Miss Florence | 1 | — | — | 1911 |
| Gidley, B. .. .. .. | 1 | — | — | 1912 |
| Goodden, H. W. .. .. | 1 | — | — | 1913 |
| Grimston, A. J. .. .. | 3 | — | — | 1921 |
| Griffiths, S. J. H. .. | 1 | — | — | 1923 |
| Griffiths, A. .. .. | 1 | — | — | 1936 |
| **H** | | | | |
| Husbands, H. Wesson | 3 | — | — | 1881 |
| Hickman, M. H. .. .. | 2 | — | — | 1881 |
| Hamilton, H. .. .. | 3 | — | — | 1883 |
| Hudson, F. H. .. .. | 1 | — | — | 1884 |
| Hartnell, G. B. .. .. | 1 | — | — | 1890 |
| Howden, P. F. .. .. | 1 | — | — | 1893 |
| Harvey, Mrs. Alfred .. | 1 | — | — | 1895 |
| Heaven, J. C. .. .. | 6 | 15 | — | 1895 |
| Hall, Miss Mabel .. .. | 3 | — | — | 1899 |
| Hawkes, Miss .. .. | 2 | — | — | 1899 |
| Hambley, Miss Elsa .. | 2 | — | — | 1903 |
| Herapath, C. E. K. .. | 5 | — | — | 1905 |
| Hull, H. R. B. .. .. | 4 | — | — | 1905 |
| Hay, A. King .. .. | 1 | — | — | 1905 |
| Hart, C. H. .. .. .. | 2 | — | — | 1906 |
| Heaven, Miss Sylvia .. | 7 | — | — | 1907 |
| Herapath, Miss N. .. | 1 | — | — | 1913 |
| Husbands, H. R. W. .. | 1 | — | — | 1914 |
| Hector, F. S. .. .. | 2 | — | — | 1920 |
| Heron, A. G. .. .. | 1 | — | — | 1920 |
| Hillman, C. .. .. .. | 1 | — | — | 1921 |
| Hughes, F. W. .. .. | 1 | — | — | 1921 |
| Ham, Miss C. I. .. .. | 5 | — | — | 1921 |
| Hooker, J. A. .. .. | 1 | — | — | 1923 |
| Hickman, Miss R. M. .. | 6 | — | — | 1924 |
| Haver, T. E. .. .. | 1 | — | — | 1925 |
| Hughes, T. I. .. .. | 1 | — | — | 1925 |
| Hill, Miss W. M. .. .. | 1 | — | — | 1928 |
| Hambley, R. C. .. .. | 4 | — | — | 1928 |
| Hughes, T. .. .. .. | 1 | — | — | 1935 |
| **I, J** | | | | |
| Jones, Miss F. C. .. .. | 7 | — | — | 1888 |
| Jones, Miss Margaret .. | 3 | — | — | 1890 |
| Iles, A. E. .. .. .. | 1 | — | — | 1906 |
| Joscelyne, Miss D. E. | 1 | — | — | 1920 |
| Jones, Miss F. M. .. | 1 | — | — | 1921 |
| Joscelyne, Miss M. V. | 2 | — | — | 1921 |
| Jackson, R. .. .. .. | 1 | — | — | 1923 |
| Jacobs, A. .. .. .. | 1 | — | — | 1923 |
| James, Miss A. D. .. .. | 3 | — | — | 1925 |
| Jackman, W. A. .. .. | — | 3 | — | 1929 |

| | Acted. | Stage Manager or Producer. | President. | Date. |
|---|---|---|---|---|
| **K** | | | | |
| Kevern, G. T. .. .. | 2 | — | — | 1892 |
| Kay-Mouat, J. R. .. | 1 | — | — | 1912 |
| Krogh, K. J. .. .. | 1 | — | — | 1926 |
| **L** | | | | |
| La Croix, W. H. .. .. | 3 | 1 | — | 1880 |
| Little, A. I. V. .. .. | 3 | — | — | 1883 |
| Lace, F. .. .. .. .. | 10 | 5 | — | 1884 |
| Lansdown, R. G. P. .. | 10 | — | 5 | 1894 |
| Lucas, J. S. S. .. .. | 2 | — | — | 1898 |
| Lee, Miss Evelyn .. .. | 1 | — | — | 1901 |
| Lee, Miss Edith .. .. | 1 | — | — | 1903 |
| Lees, Miss Duncan .. | 4 | — | — | 1910 |
| Lucas, J. V. .. .. .. | 3 | — | — | 1927 |
| Lewis, F... .. .. .. | 1 | — | — | 1929 |
| Latty, R. A. .. .. .. | 3 | — | — | 1929 |
| **M** | | | | |
| Martin, Miss Fanny .. | 1 | — | — | 1881 |
| Morton, M. F. .. .. | 1 | — | — | 1881 |
| Marsh, T. A. P. .. .. | 1 | — | 1 | 1881 |
| Meaden, E. H. .. .. | 1 | — | — | 1882 |
| Matthews, F... .. .. | 1 | — | — | 1882 |
| Marks, R. J. .. .. .. | 2 | — | — | 1884 |
| Matthews, C. .. .. | 1 | — | — | 1886 |
| Miall, C. L'O. .. .. | 1 | — | — | 1892 |
| Mountjoy, G. E. .. .. | 1 | — | — | 1895 |
| Mayo, L. .. .. .. | 2 | — | — | 1898 |
| Mitchell, C. M. .. .. | 2 | — | — | 1898 |
| Moore, L. A. .. .. | 5 | — | — | 1899 |
| Morris, L. N. .. .. | 3 | — | — | 1901 |
| Mainwaring, Miss .. | 1 | — | — | 1904 |
| Marks, R. L. .. .. | 2 | — | — | |
| Marle, S. .. .. .. | 2 | — | — | 1909 |
| Methley, Miss .. .. | 2 | — | — | 1910 |
| McCurrich, H. J. .. | 2 | — | — | 1910 |
| Moore, Mrs. Leonard .. | 1 | — | — | 1913 |
| Morris, A. H. .. .. | 1 | — | — | 1913 |
| McCurrich, Miss D. .. | 1 | — | — | 1914 |
| Moore, R. Hastings .. | 5 | 2 | — | 1926 |
| Mayer, R. G. .. .. | 1 | — | — | 1929 |
| Moore, Mrs. L. A. .. | 2 | — | — | 1935 |
| **N** | | | | |
| Nixon, J. A. .. .. .. | 3 | — | 1 | 1905 |
| Newton, Miss J. E. .. | 1 | — | — | 1928 |
| **O** | | | | |
| Openshaw, E. H. .. .. | 1 | — | — | 1888 |
| O'Leary, E. G. E. .. | 1 | — | — | 1889 |
| Ogilvy, A. .. .. .. | 1 | — | 1 | 1895 |
| Opie, P. A. .. .. .. | 4 | — | — | 1909 |
| Owen, Miss E. C. S. .. | 1 | — | — | 1929 |
| Oxley, L. R. .. .. | 1 | — | — | 1931 |
| **P, Q** | | | | |
| Penraven, A. F. .. .. | — | 1 | — | 1881 |
| Pitt, T. .. .. .. .. | 1 | — | — | 1889 |
| Peake, G. A. .. .. .. | 1 | — | — | 1892 |
| Pearse, E. M. .. .. | 3 | — | — | 1895 |
| Parkinson, G. S. .. .. | 1 | — | — | 1904 |
| Prichard, Miss Ida .. | 3 | — | — | 1905 |
| Parr, Miss M. .. .. | 3 | — | — | 1906 |
| Prichard, Miss Irene .. | 3 | — | — | 1906 |
| Peters, P. A. T. .. .. | 1 | — | — | 1912 |
| Pringle, J. A. .. .. | 1 | — | — | 1912 |
| Prichard, Miss M. .. | 1 | — | — | 1913 |
| Porter, C. P... .. .. | 2 | — | — | 1920 |

| | Acted. | Stage Manager or Producer. | President. | Date. |
|---|---|---|---|---|
| PULLEN, Miss D. M. .. | 2 | — | — | 1921 |
| DE LA PASTURE, Miss Y. | 3 | — | — | 1922 |
| PENGLAZE, A. J. .. .. | 3 | — | — | 1922 |
| PERRY, C. R. .. .. .. | 3 | — | — | 1922 |
| POSTHUMAS, Mrs. .. .. | 1 | — | — | 1923 |
| PRICE, D. A. C. .. .. | 3 | — | — | 1924 |
| PROWSE, A. S. .. .. | 1 | — | — | 1924 |
| POTTER, Miss M. F. .. | 1 | — | — | 1927 |
| PAULI, C. H. .. .. .. | — | 2 | — | 1931 |
| PHILLIPS, P. .. .. .. | 2 | — | — | 1935 |
| **R** | | | | |
| RIDDELL, A. W. .. .. | 1 | — | — | 1881 |
| RUSSELL, Mrs... .. .. | 1 | — | — | 1886 |
| RENNY, Miss .. .. .. | 1 | — | — | 1892 |
| ROSE, Miss Alice .. .. | 6 | — | — | 1895 |
| ROSE, Miss J. .. .. | 3 | — | — | 1895 |
| ROGERS, Mrs. F. W. .. | 7 | — | — | 1898 |
| RUDGE, F. H. .. .. | 2 | — | — | 1899 |
| RITBLAT, L. R. .. .. | 3 | — | — | 1920 |
| REYNOLDS, Miss H. E. | 2 | — | — | 1920 |
| ROWLEY, A. T. F. .. | 1 | — | — | 1923 |
| RILEY, Iona .. .. .. | 1 | — | — | 1928 |
| RICHARDSON, J. S... .. | 1 | — | — | 1935 |
| **S** | | | | |
| SPENCER, H. A. .. .. | — | — | — | 1880 |
| SALMON, L. E. A. .. .. | — | — | — | 1881 |
| SIMMONS, H. .. .. .. | 1 | — | — | 1881 |
| SMITH, Miss F. .. .. | 2 | — | — | 1882 |
| SMITH, G. Munro .. .. | 11 | — | 8 | 1882 |
| STEPHENSON, C. H. .. | — | 1 | — | 1882 |
| SAINSBURY, Miss .. .. | 1 | — | — | 1884 |
| SIMONS, R. J. R. C. .. | 2 | — | — | 1884 |
| SHEPPARD, Miss G. .. | 2 | — | — | 1888 |
| STACY, Miss .. .. .. | 3 | — | — | 1890 |
| SWAIN, James .. .. | 8 | 3 | — | 1890 |
| SHAW, J. E. .. .. .. | — | — | 2 | 1891 |
| STACY, Miss Trixy .. | 1 | — | — | 1891 |
| STEELE, Miss .. .. .. | 1 | — | — | 1893 |
| STEELE, Miss S. .. .. | 2 | — | — | 1893 |
| STOCK, S. V. .. .. .. | 12 | 6 | — | 1894 |
| STOCK, P. G. .. .. .. | 1 | — | — | 1895 |
| STOCKWELL, R. .. .. | 1 | — | — | 1898 |
| SANDERSON, Alan .. .. | 1 | — | — | 1900 |
| SMITH, L. Shingleton .. | 2 | — | — | 1905 |
| SHEPPARD, A. L. .. .. | 1 | — | — | 1906 |
| SMITH, R. Shingleton .. | — | — | 3 | 1906 |
| STACK, E. H. E. .. .. | 3 | 2 | — | 1906 |
| SYLVESTER, Miss H. .. | 1 | — | — | 1907 |
| SMYTHE, H. J. Drew .. | 3 | — | — | 1910 |

| | Acted. | Stage Manager or Producer. | President. | Date. |
|---|---|---|---|---|
| SCOTT, Miss .. .. .. | 1 | — | — | 1912 |
| STOCK, Mrs. S. V. .. .. | 1 | — | — | 1914 |
| SPENCER, G. .. .. .. | 1 | — | — | 1914 |
| STATHAM, R. S. S. .. .. | — | 1 | — | 1914 |
| SIEPMAN, Miss P. .. .. | 1 | — | — | 1920 |
| SYMES, J. O. .. .. .. | — | — | 1 | 1921 |
| SEVILLE, H. E. D... .. | 1 | — | — | 1928 |
| STOCK, J. P. P. .. .. | 1 | — | — | 1928 |
| SMYTHE, Mrs. Drew .. | 2 | — | — | 1935 |
| **T** | | | | |
| TREVELT, Miss A. .. .. | 1 | — | — | 1881 |
| TRASK, J. E. .. .. .. | 1 | — | — | 1884 |
| TAYLOR, W. E. .. .. | 1 | — | — | 1909 |
| TERRELL, Miss C. M. .. | 1 | — | — | 1921 |
| TAYLOUR, Miss E. A. .. | 1 | — | — | 1929 |
| TAYLOR, T. H. .. .. | 1 | — | — | 1929 |
| **U, V** | | | | |
| VILLIERS, Mrs. .. .. | 1 | — | — | 1886 |
| **W** | | | | |
| WETHERED, F. J. .. .. | 2 | — | 4 | 1881 |
| WEATHERLY, E. .. .. | — | — | 1 | 1883 |
| WEATHERLY, Lionel .. | 1 | 2 | 1 | 1883 |
| WARD, E... .. .. .. | — | — | 1 | 1884 |
| WOOD, Miss F. .. .. | 1 | — | — | 1884 |
| WRIGHT, W. Southey .. | 3 | 1 | — | 1888 |
| WEBBER, Miss Ella .. | 1 | — | — | 1892 |
| WILLIAMS, F. D'Arcy .. | 2 | — | — | 1892 |
| WILTON, Mrs. .. .. .. | 1 | — | — | 1893 |
| WOOD, C. G. Russ.. .. | 1 | — | — | 1893 |
| WEBB, W. T. .. .. .. | 6 | — | — | 1899 |
| WALDO, Cecil .. .. .. | 1 | — | — | 1900 |
| WEATHERLY, Miss Muriel | 1 | — | — | 1900 |
| WALTERS, C. F. .. .. | 4 | — | — | 1901 |
| WEDMORE, J. C. .. .. | 1 | — | — | 1903 |
| WRIGHT, A. J. .. .. | 1 | — | — | 1903 |
| WALTERS, Miss Alice .. | 2 | — | — | 1908 |
| WALMSLEY, B. N. .. .. | 1 | — | — | 1909 |
| WALDO, Miss I. .. .. | 1 | — | — | 1911 |
| WHICHER, Miss .. .. | 1 | — | — | 1913 |
| WILLIAMS, P. Watson .. | — | — | 1 | 1914 |
| WATSON, Miss K. .. .. | 2 | — | — | 1921 |
| WALKER, Cyril .. .. | — | — | 1 | 1922 |
| WILKINSON, Miss M. E. G. | 2 | — | — | 1923 |
| WEIR, Miss S. .. .. | 1 | — | — | 1925 |
| WALTERS, M. .. .. .. | 2 | — | — | 1935 |
| **X, Y, Z** | | | | |
| YORK, Mrs. .. .. .. | 1 | — | — | 1896 |

